# Quantifying the energy and emissions implications of consumption redistribution in the UK through sustainable consumption corridors

**DOI:** 10.1038/s41598-025-01495-0

**Published:** 2025-05-12

**Authors:** Sam Betts-Davies, Anne Owen, John Barrett, Paul Brockway, Jonathan Norman

**Affiliations:** 1 Sustainability Research Institute, School of Earth and Environment, University of Leeds, Woodhouse Lane, Leeds, LS2 9JT UK; 2 Energy Demand Research Centre, Jubilee Building, University of Sussex Business School, University of Sussex, Falmer, Brighton, BN1 9SL UK

**Keywords:** Climate-change mitigation, Socioeconomic scenarios, Sustainability, Energy justice

## Abstract

Reducing inequality to ensure decent living standards alongside climate mitigation, are frequently posited as dual goals of a just transition. Energy sufficiency has received attention as a solution to these crises, but there has been limited exploration of the impact sufficiency principles could have on energy and GHG emissions. Addressing this gap, we utilise a consumption-corridor approach to develop three redistributive scenarios of final consumption for the UK. Each scenario ensures all households meet essential needs and facilitates social participation, whilst varying the level of consumption redistribution. These scenarios are modelled using the UK Multi-Regional Input Output model to estimate the impact of redistribution on the consumption-based energy and emissions footprints of 13 household types. We find that reductions in consumption inequality can support reductions in GHG emissions and energy use, but only if the consumption of higher-income consumers is limited to near equality in expenditure. We also find significant shifts in the composition of consumption resulting from redistribution that may support climate mitigation, such as reduced car use and flying, particularly in scenarios where higher household consumption is curtailed. We conclude that economic inequality must be integrated into climate mitigation modelling to develop effective solutions.

## Introduction

### Energy demand reduction is essential for climate change mitigation

Reducing global energy demand is essential to de-risking the climate mitigation targets outlined in the Paris Agreement^1^. Demand reduction carries fewer environmental risks than supply-side technologies^2^, it reduces reliance on speculative future capabilities of carbon dioxide removal technologies (CDR)^3–6^, and can support a high or improved quality of life. It is also essential to national mitigation pathways, particularly in wealthier nations with high energy use. In the UK, a review of mitigation scenarios suggested that, at a minimum, energy demand in 2050 would have to be reduced by at least 40% to achieve climate targets without unrealistic levels of CDR^7^, with others indicating reductions of up to 50% could be desirable^8^. Whilst essential to climate and ecological goals and capable of increasing wellbeing, if energy demand reduction is enacted without attention to social or energy justice, interventions could negatively impact levels of inequality and wellbeing in society^9^.

### Considering inequality in the climate mitigation transition

Climate and energy justice have risen to the fore of decarbonisation debates^10^, with many now exploring the inequality in global and national distributions of responsibility for both historic and present levels of energy use^11^ and emissions^12^. Similar discussions are found in public-facing debates, following the publication of Oxfam’s report^13^ on climate inequality, highlighting the impact of the overconsumption of the worlds richest 1%, whose responsibility equates to the same level of emissions as the poorest 66% of the global population.

This is in a context where no countries have yet achieved all important social thresholds in meeting the needs of citizens within biophysical resource constraints^14^. Some argue through reducing excessive consumption of wealthier groups, space within ecological constraints can allow access to important energy services to increase and fulfil decent living standards universally^9,15–19^, while living within planetary boundaries^20.^ This imperative to reduce economic and energy inequality crosses opposed narratives of climate mitigation^21^, with its inclusion in green growth^22,23^, the Green New Deal^24^ and degrowth literature^19,25,26^.

Literature examining the impact of inequality on the environment is not lacking. Hailemariam et al.^27^ review empirical analyses of income inequality’s impact on carbon emissions, finding mixed evidence on the direction of this relationship in the literature. Some studies find that lower inequality causes a downward pressure on emissions^28^, others suggest inequality reduction supports the decoupling of GDP and emissions, particularly in high-income countries^22^. Conversely, some find that the lower marginal propensity to consume of wealthier households leads to emissions growth when income is made more equal^27,29^.

### The need to ensure minimal needs are achieved

Whilst these studies examine statistical relationships between inequality and emissions across spatial and temporal contexts, many overlook the core purpose of inequality reduction: securing acceptable living standards for all. When assessing observed links between inequality and emissions, studies exploring this statistical relationship rarely consider how reducing inequality affects the attainment of decent living standards. This highlights the need to examine disparities in energy use, as access to energy services through goods and services underpins minimal wellbeing^30^.

There is literature examining minimum living standards as an important factor. At present levels of energy inequality across Europe, Jaccard et al.^15^ find that achieving 1.5°C requires significant levels of CDR, large efficiency improvements and a low minimum final energy use. Increasing this to a realistic level requires close to full equality. Millward-Hopkins^16^ comes to similar conclusions at a global level, finding that inequality substantially increases the energy needed to ensure decent living universally. Without inequality reduction, even more ambitious levels of renewables and CDR are necessary to achieve mitigation goals alongside decent living standards.

This literature predominantly focuses on inequality, energy and living standards at a continental or global scale^15–17,31,32^. Given inequalities in consumption, energy, and responsibility for emissions are at their widest globally, international inequality reduction is imperative to ensure climate justice. However, as inequality and climate mitigation policies are implemented nationally, international-scale research is either difficult to translate into policy or must be disaggregated to nation-states. Defining the energy or resource use needed for universal human needs at a global level is important^31–33^. However, in practice, these needs—and the energy required to meet them—vary across countries due to geographical and climatic conditions, socio-cultural norms, public service provision, infrastructure, and societal perceptions of decent living. Therefore, research on national inequality reduction, grounded in nationally defined minimum consumption levels, and its implications for energy use and climate targets is essential.

## Research design

This study’s methodology is fully detailed in the "Methods" section; however, it is worth briefly outlining the study’s design, key aims, data sources, and scenario construction here. The study aims to empirically model the impact of consumption redistribution scenarios on UK energy demand and emissions. The UK was chosen due to the availability of highly disaggregated expenditure data for households^34^, the highly developed reference budgets describing a socially acceptable expenditure profile required for a minimally decent life in the UK^35^, and a leading input/output modelling framework (UKMRIO) to derive energy and emissions footprints from redistributed expenditure scenarios^36^.

The research questions guiding this study include:

1. How is expenditure currently distributed across UK households and how does this relate to households’ abilities to meet their needs? (Ssee "Some households’ consumption distributions are more unequal than others.")
2. What is the consumption-based energy and emissions implications of consumption levels associated with a minimum acceptable standard of living? (see "MIS scenario")
3. How could household consumption redistribution impact the United Kingdom’s consumption-based energy and GHG emissions footprints? (see "Consumption-based energy use and GHG footprints of redistributive scenarios")
4. How does redistribution impact the structure of the UK’s energy demand and emissions, and how does this impact the wider mitigation challenge? (see "Consumption-based energy use and GHG footprints of redistributive scenarios" and "Pursuing redistribution in support of climate mitigation")

**Fig. 1:**
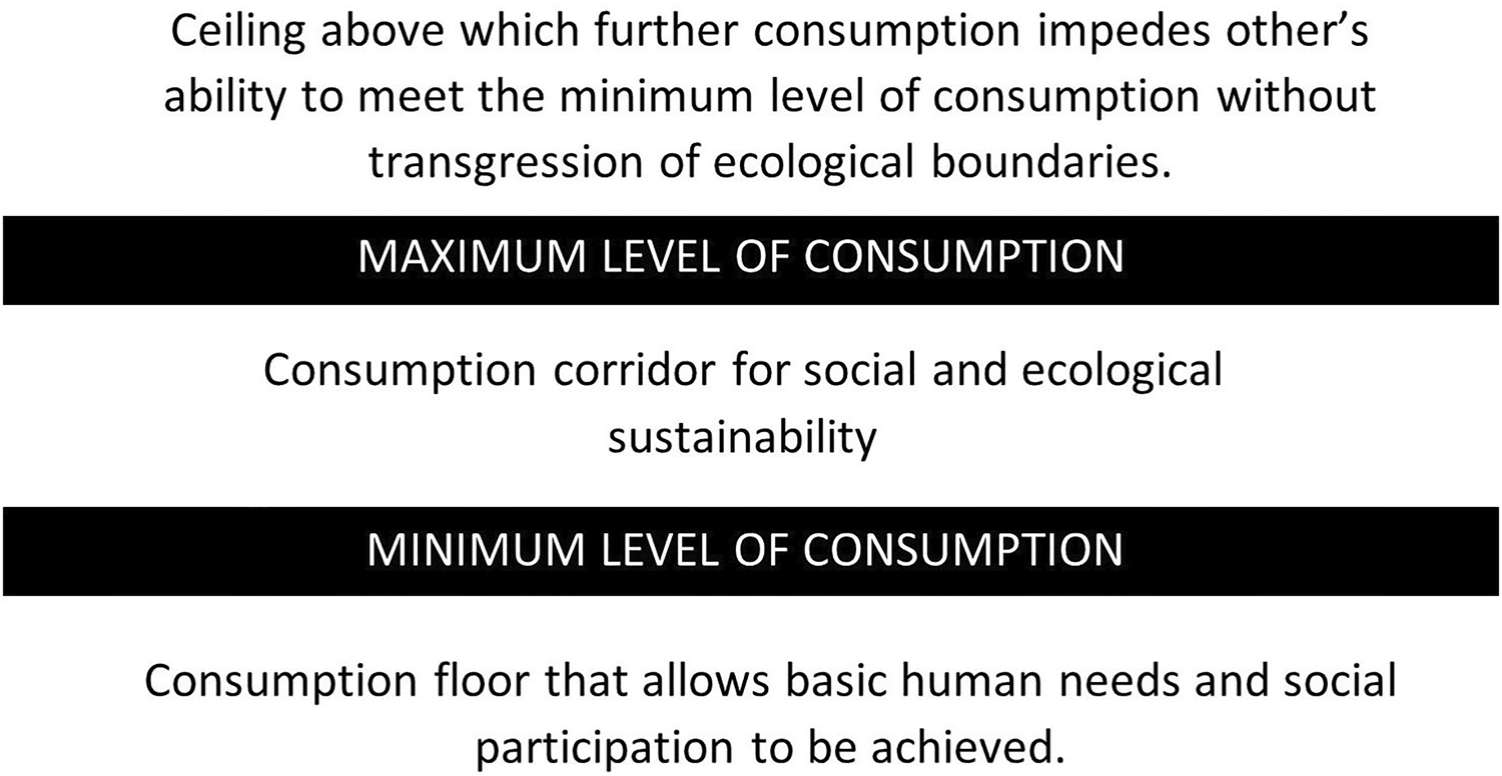
Graphical depiction of consumption corridors adapted from Fuchs et al.^41^.

We construct static consumption scenarios that model the impact of consumption redistribution on consumption-based UK energy demand and GHG emissions, utilising a consumption corridor approach. Consumption corridors, illustrated in Fig. 1, describe a minimum standard that fulfils social needs whilst remaining within a maximum consumption level set by an ecological ceiling^37–40^. This ceiling defines the limit above which further consumption threatens the ability of others to consume the minimum level of consumption, given the biophysical constraints to resource consumption defined by planetary boundaries^41^.

The consumption corridor concept shares similarities to other strands of post-growth economics, such as doughnut economics and the wellbeing economy. Each emphasise the importance of ensuring everyone can live in comfort and safety, and view the economy’s purpose as distributing resources to achieve this while enabling ecological sustainability^42,43^. While doughnut economics and the wellbeing economy operate at a broader macro-level, addressing foundational issues beyond resource consumption, such as social networks, communities, political voice, education, and technology^42,43^—consumption corridors focus more directly on the resource use required to meet material and social needs. This makes the concept particularly useful for developing redistributive consumption scenarios in sustainability contexts.

As noted, prior studies on the inequality–climate nexus have either: (a) not explicitly linked redistribution to minimum consumption needed to meet social needs^18^; (b) included arbitrarily defined social floors, often based on income ratios^15,17^; or (c) applied universal needs-based approaches, like the ‘Decent Living Standards’ framework^31–33^, rather than nationally defined minimum consumption budgets. The consumption corridor approach addresses these gaps by (a) tying redistribution to meeting minimum needs through a non-arbitrary social floor, and (b) allowing for design sensitive to national contexts affecting need satisfaction.

To develop redistributive scenarios that resolve this gap, we employ a reference budget approach to defining a minimum consumption level. Grounded in socio-economic research of poverty lines and living wages, reference budgets utilise a qualitative research methodology involving focus groups with the public and interviews with experts, to determine a socially acceptable basket of goods defined as the minimum expected consumption level^44,45^. The UK has rich and detailed pre-existing research developing annual reference budgets for 13 household types in the UK (~ 86% of all UK households) called the Minimum Income Standard (hereafter referenced as MIS)^35,46^.

Based on participatory focus groups and expert guidance, this MIS-derived minimum consumption level represents what the UK public believes is necessary for households to fulfil their needs, live with dignity, and fully participate in society. It’s a higher social floor than requirements to meet basic needs, such as the ‘decent living standards’^31–33^. Rather, the MIS describes an ‘acceptable standard of living’ beyond ‘just food, clothes and shelter’, to include ‘what you need to have the opportunities and choices necessary to participate’ in UK society^46^. In practice, this produces highly disaggregated baskets of goods for 13 household types that can be used to define a floor in consumption.

**Fig. 2:**
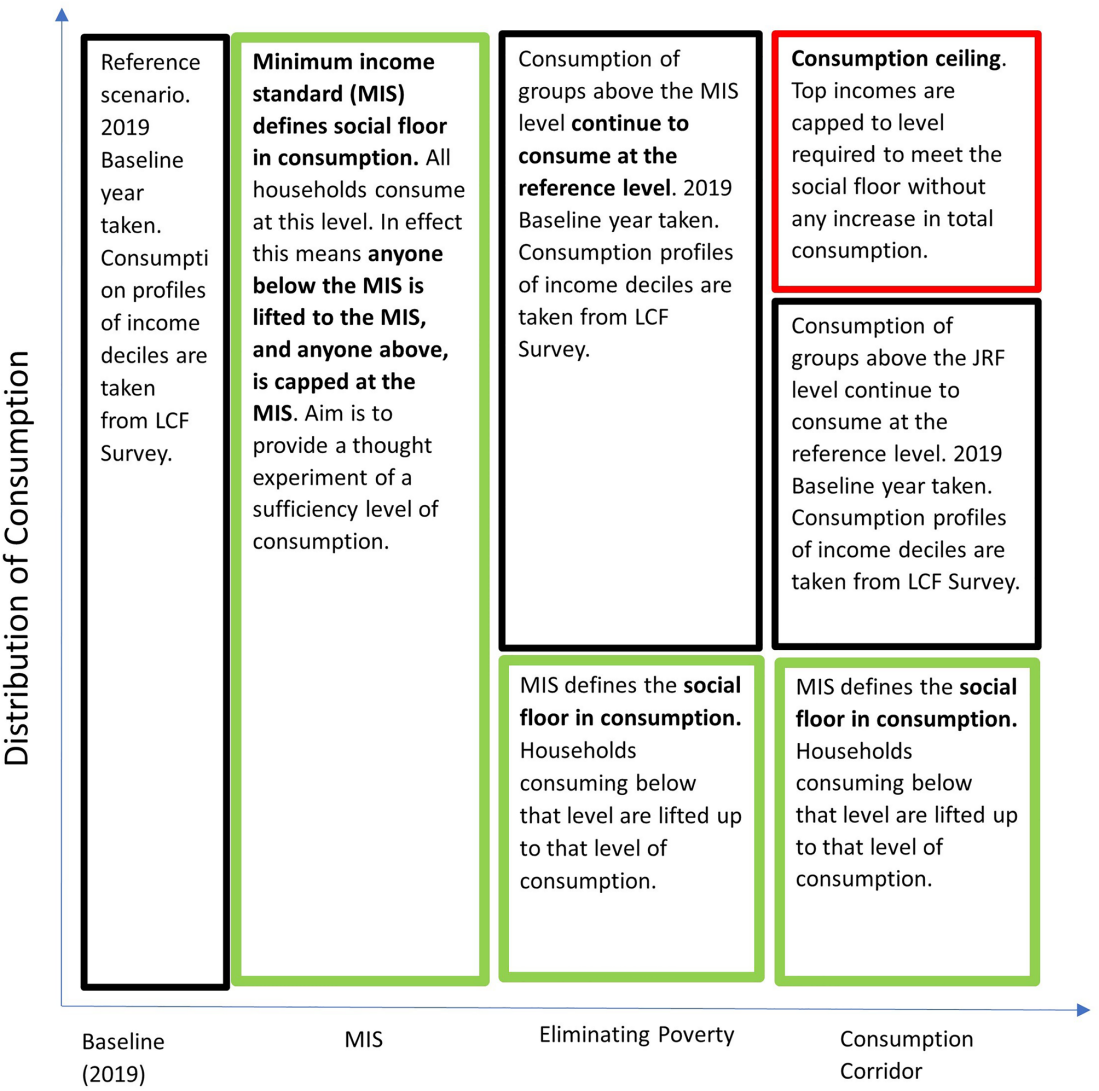
Overview of final demand scenarios (y axis not to scale). The scenarios represent consumption corridors, with the boxes in green representing a raising of consumption levels that fall below a minimum level defined by the MIS Minimum income standard. The red boxes represent caps on consumption consistent with a consumption ceiling. The black boxes represent households that sit between the consumption floor and consumption ceiling that are left unchanged.

The emissions footprint of the MIS budgets has previously been modelled using an input–output model in 2010, using MIS budgets from 2008 and final demand and emissions data from 2004^47^. Described as an egalitarian ‘reduced consumption scenario’, where everyone in the UK consumes at the MIS, it was found to result in a 37% reduction in consumption-based GHG emissions. The MIS scenario, described in Fig. 2, updates this analysis using 2019 data, reflecting the changes in the requirements for a decent life, changes in GHG emissions intensities, and increased model resolution, improving the accuracy and richness of the results.

This analysis further advances previous studies by examining the effects of redistribution on both consumption-based energy use and GHG emissions. As energy use underpins living standards and serves as a key climate policy lever, understanding the impact of achieving minimum consumption levels on energy demand is crucial. In addition, two further scenarios are developed using the consumption corridor framework (Fig. 2). The *Eliminating Poverty* scenario raises those below the MIS to meet essential needs, while the *Consumption Corridor* scenario adds an expenditure cap. Although not directly linked to an ecological ceiling, this expenditure cap limits overconsumption to the level of redistribution required to universally meet the social floor, thereby limiting the ecological impact of needs satisfaction. This adaptation of the original consumption corridor concept that utilises ecological limits on consumption (Fig. 1) is discussed fully in the methodology (see "Scenario 3: consumption corridor scenario"). Full narrative and methodological details for all scenarios are provided in the"Methods".

## Results

### Some households’ consumption distributions are more unequal than others

**Fig. 3:**
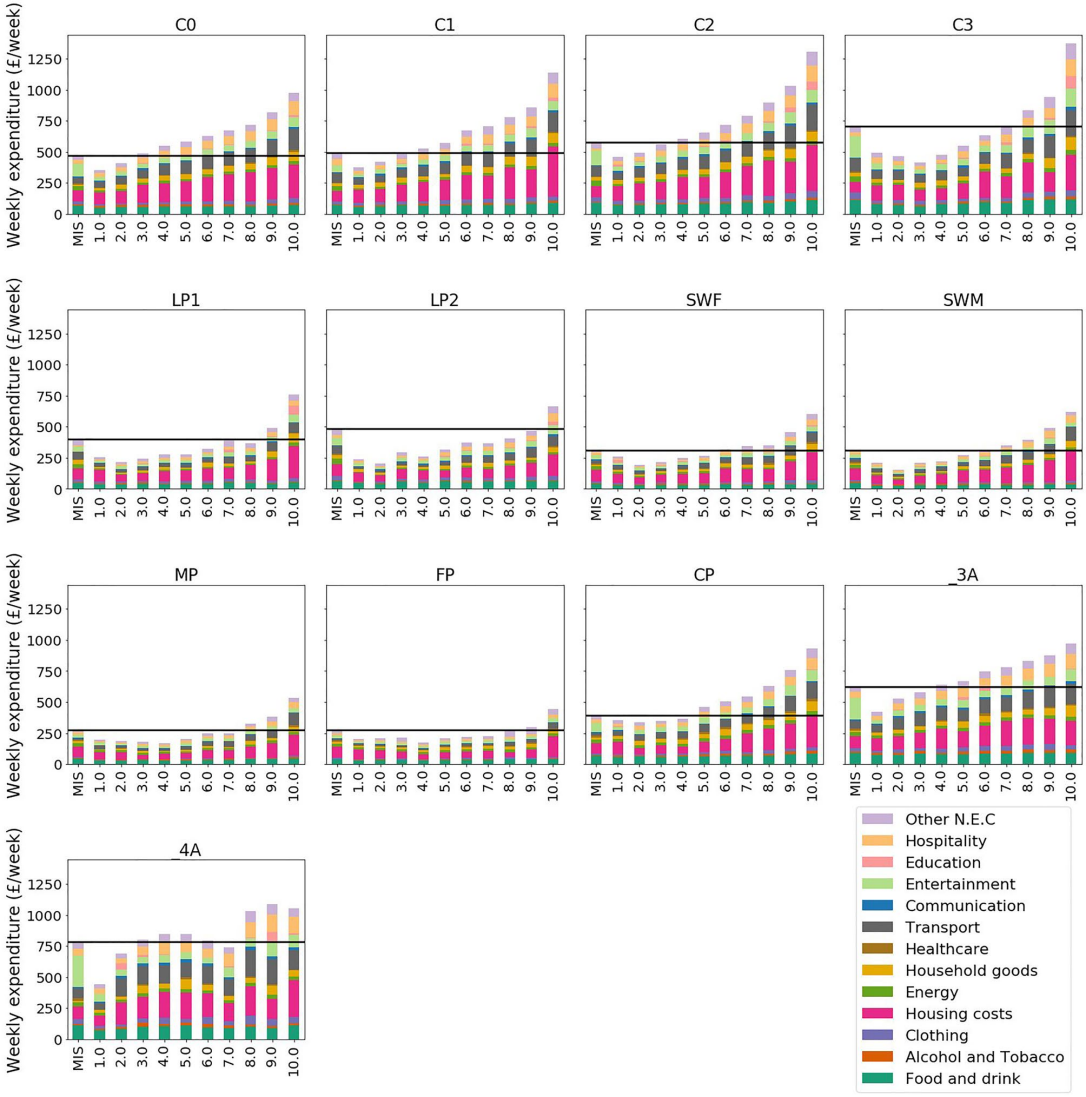
Stacked bar chart comparing the MIS minimum weekly consumption level with the average household consumption patterns split by household type and income decile. The first bar and black line across each plot represents the total MIS consumption level for each household type. Household type key: C0 = Couple (21.4% of households included in the study), C1 = Couple + 1 child (8.6% of households), C2 = Couple + 2 (9.7% of households), C3 = Couple + 3 (2.3% of households), LP1 = Lone parent + 1 child (2.6% of households), LP2 = Lone parent + 2 (2.3% of households), MP = Male Pensioner (5% of households), FP = Female Pensioner (9.5% of households), CP = Coupled Pensioners (14.5% of households), _3A = 3 Adults (6.6% of households), _4A = 4 adults (2.4% of households), SWM = Single Working Age Male (8.6% of households), SWF = Single Working Age Female (6.3% of households).

Addressing RQ1 on the distribution of household consumption across types, Fig. 3 shows the MIS budgets within current consumption distributions for each household type, divided by income deciles. It highlights expenditure inequality within household types and their consumption relative to the MIS minimum, revealing that consumption inequality and needs satisfaction are not evenly distributed.

Addressing consumption inequality, the ratio between the 10th and 1st income deciles varies across household types. For example, couples with one child (C1) have a 90/10 expenditure inequality ratio of 3:1, while the female pensioner (FP) household type has a ratio of 2:1, indicating less inequality.

This inequality across household types affects their ability to meet needs, as shown by the MIS bar and horizontal line in Fig. 3. The position of this line within the income distribution highlights the significant need in the UK to increase consumption for many households to ensure access to basic needs and societal participation. However, this need varies across household types. For coupled households with zero to two children (C0, C1, C2), 20–30% consume below their MIS, while many consume well above it. In contrast, 90% of lone parent households with two children (LP2) consume below their MIS. High underconsumption is also found in lone parents with one child (LP1) and female pensioners (FP), with 80% of these households consuming below the MIS.

This expenditure inequality, relative to households’ varying need satisfaction, underscores the importance of this approach in designing fair redistributive scenarios for sufficient consumption. The analysis suggests that the greatest overconsumers are not necessarily the highest consumers or earners, but those who exceed their relative needs the most. While 4 adult households (4A) are high absolute consumers, higher-income couples without children (C0) are more likely to overconsume relative to their needs. Understanding household needs and their variations is crucial for ensuring that consumption corridors enable just and effective redistribution.

### Consumption-based energy use and GHG footprints of redistributive scenarios

**Fig. 4:**
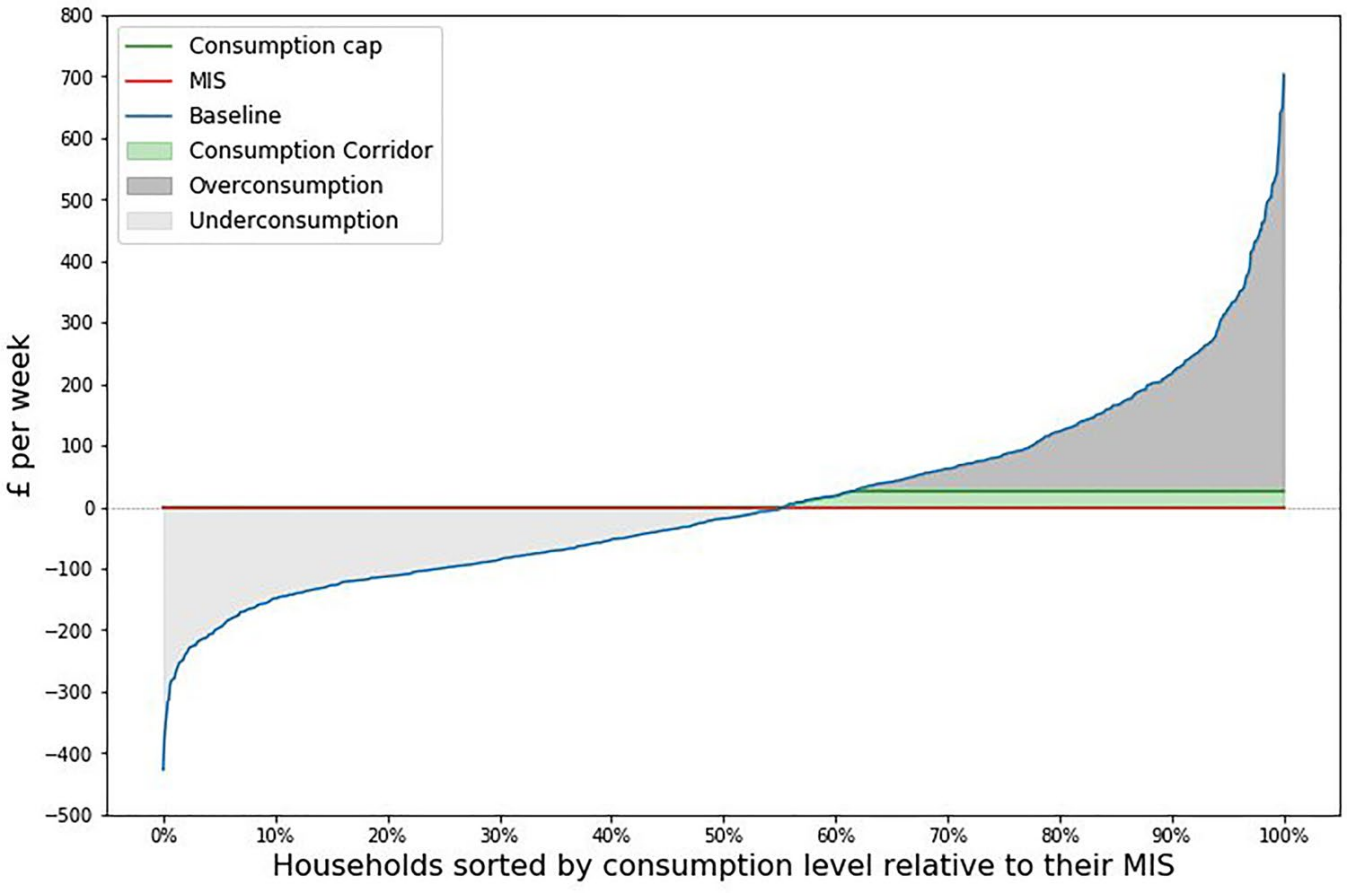
Line chart showing the consumption of households relative to their household type’s need satisfaction point (i.e. their MIS budget). Households are sorted from most under consuming relative to their MIS, to most over consuming relative to their MIS. 0 on the y axis in each subplot is consumption at the MIS budget. The blue line represents the current consumption distribution of households, i.e. the Baseline scenario. The orange line, at y = 0 represents the MIS scenario. The light grey shaded area labelled as underconsumption represents all those households in the baseline that are consuming beneath their MIS budget. The green line represents the consumption cap, as calculated in the consumption corridor scenario (see methods section "Scenario 3: consumption corridor scenario"). Consumption above this line, shaded in dark grey represents over consumption, and the green shaded area, between the MIS and the consumption cap, represents the consumption corridor.

Fig. 4 displays household expenditure relative to each household’s need satisfaction consumption level (total weekly consumption – MIS budget). The lowest consuming household relative to needs is positioned on the far left of the x-axis, and the highest on the far right. The baseline (blue) shows the current distribution’s ability to meet household needs. Underconsumption is represented by the light grey area between the baseline and the MIS scenario (orange). The *Eliminating Poverty* scenario lifts households up to the MIS, while allowing those above the MIS to remain at their current level. The *Consumption Corridor* scenario introduces a consumption cap (green line), where financial transfers fund the increase in expenditure to eliminate underconsumption. The dark grey area above this cap represents overconsumption. As described in the methodology (see "Scenario 3: consumption corridor scenario"), the cap is only £25.99 per week higher than each household type’s MIS, resulting in a very equal consumption profile.

While the consumption corridor profile and the MIS appear similar, their expenditure composition differs significantly. The reduction in expenditure for households affected by the consumption cap is distributed according to consumption category elasticities, rather than the prioritised areas in the MIS budgets. This is further elaborated on when discussing the scenario footprints’ impacts.

**Fig. 5:**
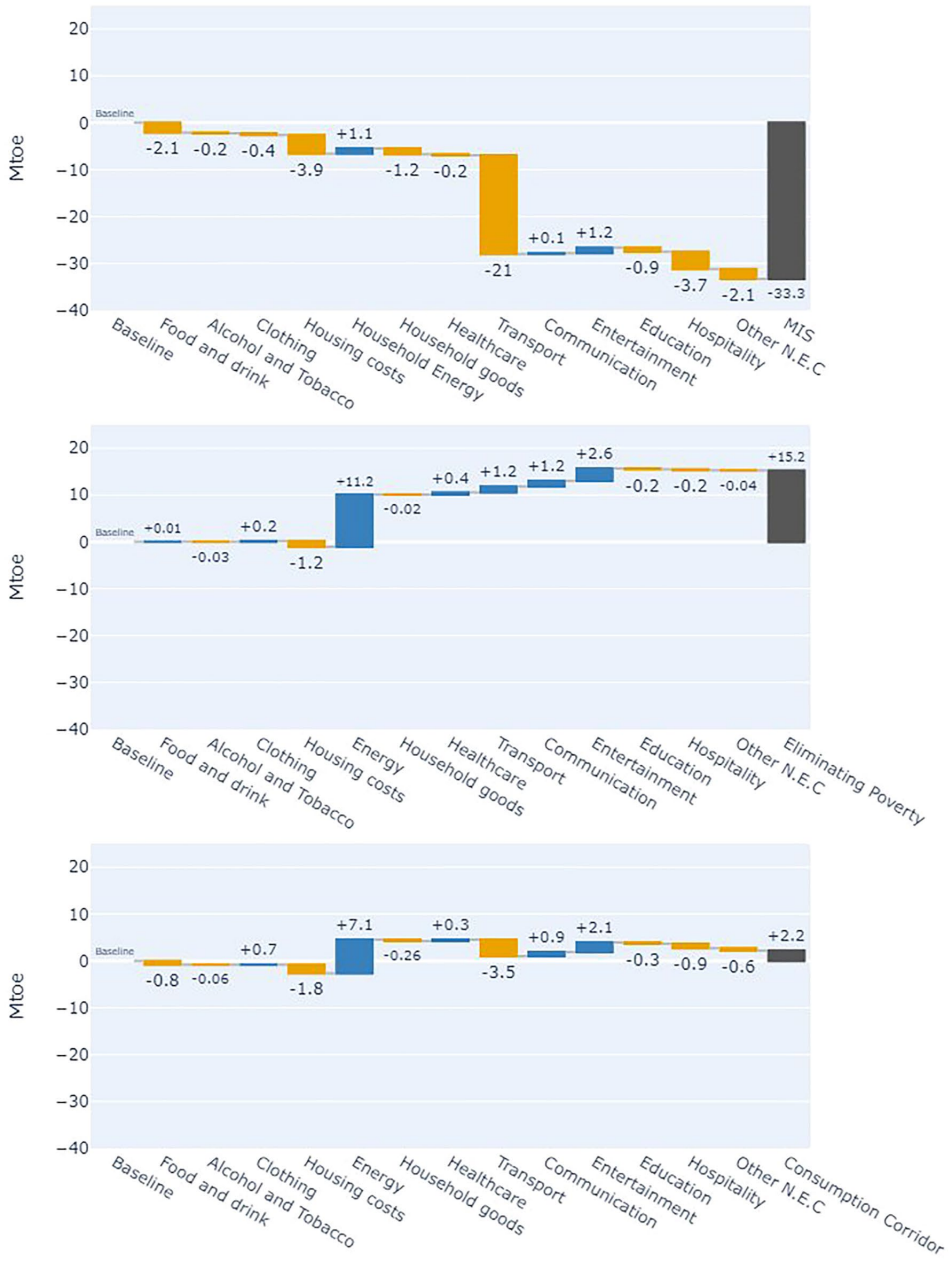
Waterfall plot showing the contribution to change in the annual consumption-based energy use footprints (Mtoe) based on (top) the MIS scenario, (middle) the Eliminating Poverty scenario, and (bottom) the Consumption Corridor scenario. Yellow bars represent a reduction in energy use in a category, blue bars represent an increase, and the black bars represent total change in the energy use footprint of the scenario.

**Fig. 6:**
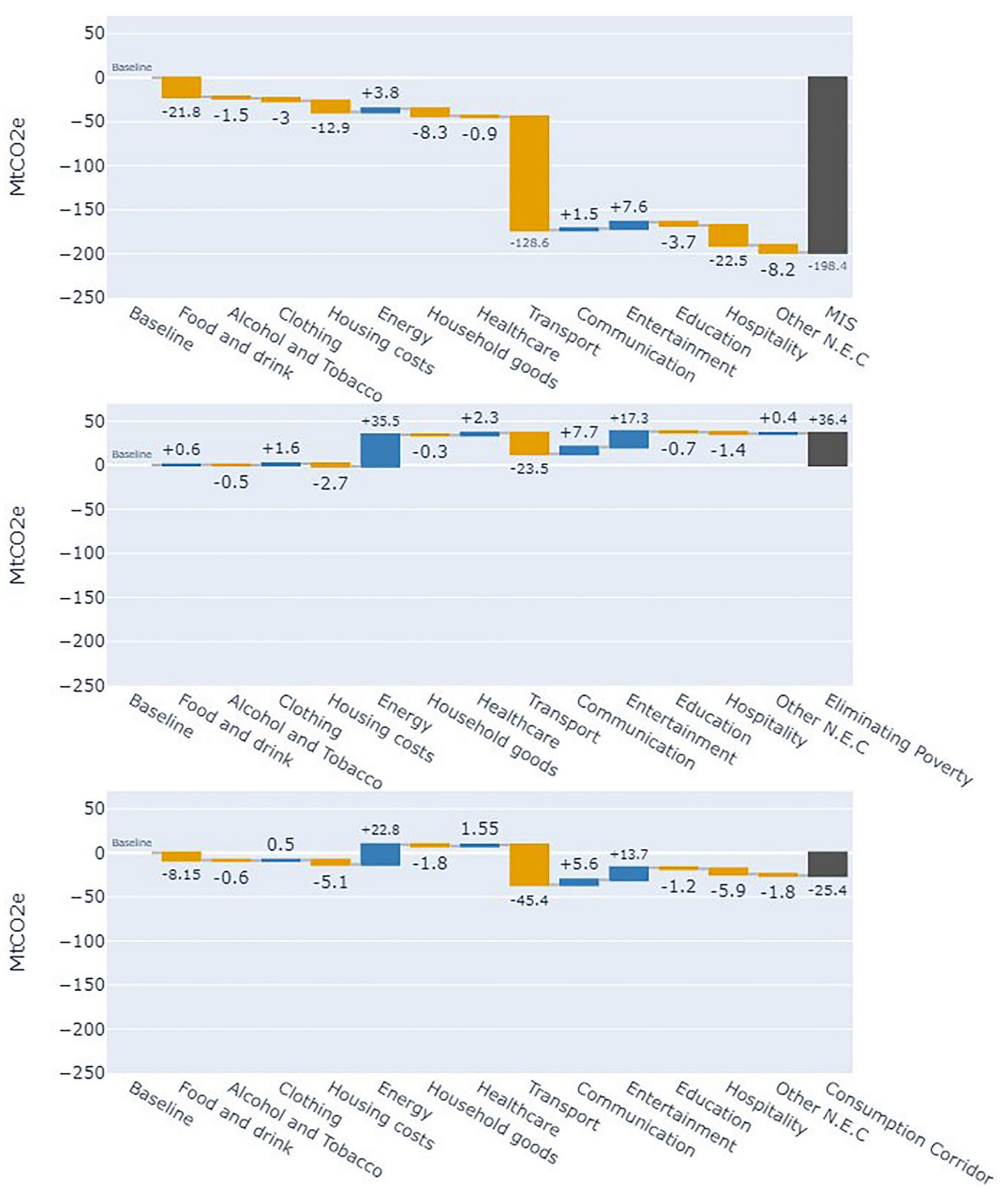
Waterfall plot showing the contribution to change in the annual Consumption based greenhouse gas emissions (MtCO2e) based on the MIS scenario (top), the Eliminating Poverty scenario (middle), and the Consumption Corridor scenario (bottom). Green bars represent a reduction in emissions in an expenditure category, red bars represent an increase, and the blue bars represent total change in the GHG footprint of the scenario.

Addressing RQ2 and RQ3, Figs. 5 and 6 are waterfall plots showing changes in consumption-based energy (Fig. 5) and GHG emissions (Fig. 6) footprints across redistributive scenarios. Each plot displays the absolute change in energy and emissions footprints across 13 expenditure categories relative to the baseline, with the final bar representing the aggregate footprint of each scenario.

#### Minimum Income Standard (MIS) scenario

The MIS scenario shows the largest reductions in energy and emissions footprints, with the energy footprint falling by 31% and GHG emissions footprints by 39% compared to the baseline, despite only a 2.3% reduction in aggregate household expenditure. Most savings stem from shifts in expenditure towards categories identified by the MIS as essential for meeting needs and social participation. These savings indicate that non-prioritised categories have significantly higher intensities per £ of expenditure. The expenditure shifts are detailed in supplementary note 1.

The largest reductions occurred in transport, with energy footprints reduced by 21 Mtoe and emissions by 128.6 MtCO2e, driven by the significant decrease in car ownership in the MIS, limited to households with children. This results in an 81% reduction in consumption-based GHG emissions and energy use related to vehicle expenditure. Similarly, the MIS does not identify a minimum need for holidays abroad, eliminating spending on aviation and saving additional energy and emissions. This shift to domestic holidays leads to a 136% increase in the carbon and energy footprint of public transport.

The generalisability of these household budgets (and their footprints) is weakened by their inability to account for the specific needs of individual households. In the case of car use, there will be circumstances where households have a legitimate need for a car, i.e. due to geographical, health or disability related needs, without children residing in the property^48^. These cases are omitted and would lead to increased car use. Despite this, the reduction in car use is consistent with studies of redistribution at a global level, which observe reductions in transport when expenditure is made more equal^18^.

#### Eliminating poverty scenario

In the *Eliminating Poverty* scenario, where households below the MIS are raised to that consumption level, the energy footprint of household expenditure increases by 14%, and consumption emissions by 7%. This results from a 12% rise in total household expenditure, driven primarily by increased home energy use among households now consuming at the MIS. The energy footprint of home energy use rises by 11.2 Mtoe, accounting for nearly ¾ of the total energy increase.

This rise in home energy use is caused by the heating regime prescribed by the MIS. Whilst not explicit in recent reports, past MIS reporting (confirmed still to be the case by the MIS research team) suggests the budgets for heating are calculated using WHO-recommended internal temperatures of 20–21°C, with heating regimes between 6 h for working adults and 16 h a day for households with older or younger inhabitants^47,49^. This heating regime is significantly greater than is represented in the baseline.

The average heating regime in the UK was found to be only 7.5 h a day, with the average thermostat set to 20^o^C^50^. In households consuming at the MIS, the weighted average time a dwelling is heated for is 12.4 h a day, representing a significant increase in heating use compared with the baseline and a rise in energy use. However, given that in 2022, 13% of households lived in energy poverty (Low-income low energy efficiency definition)^51^, and 30% of households spend more than 10% of their disposable income after housing costs on energy, part of this increase in energy use is accounted for by facilitating all to heat their home sufficiently.

#### Consumption corridor scenario

In the *consumption corridor* scenario, where a consumption cap enables those under the minimum consumption level to consume at the MIS without increasing aggregate consumption, the footprint results differ across energy demand and emissions. The scenario yields a 2% increase in the energy footprint, due to the rise in household energy use as previously described, only being somewhat tempered by the reduction of those above the cap.

Comparatively, the consumption-based GHG emissions are reduced by 5%, despite aggregate expenditure remaining constant. Here, a shift in expenditure from transport towards home heating represents a shift from higher GHG-intensive sectors, such as the lifecycle emissions from car use and air travel, towards the less intensive household energy.

Comparing this scenario to the MIS addresses RQ4 by examining the structure of UK energy demand and emissions. Although aggregate expenditure in the consumption corridor scenario is only 2% higher than in the MIS, its energy footprint is 50% higher and its emissions footprint 60% higher. This is because households impacted by the cap reduce consumption following current expenditure trends rather than the MIS-prioritised categories. Households within the corridor remain unchanged, and reductions from the cap are distributed across categories using elasticity values, reflecting each category’s sensitivity to changes in total consumption (see methods section "Scenario 3: consumption corridor scenario").

As a result, shifts from higher- to lower-impact categories are less pronounced than in the MIS scenario. For instance, the MIS scenario shows a 73% reduction in car-related spending, a 100% drop in aviation, and a threefold increase in public transport expenditure. In contrast, the consumption corridor yields only a 33% reduction in car spending, 41% in flights, and a 55% rise in public transport. These differences in the distribution of high-consuming households’ expenditure patterns across consumption categories explain the divergence in energy and emissions footprints, highlighting the potential importance of redirecting spending toward lower-impact need-focused categories alongside reducing aggregate levels of consumption (see supplementary note 1).

## Discussion

### Pursuing redistribution in support of climate mitigation

Achieving acceptable minimum standards of living whilst ensuring a transition to net-zero is at the heart of just transitions, and many economic narratives of climate change mitigation^21^. Through modelling the energy and emissions implications of a range of expenditure profiles for the UK, this study has attempted to understand the extent and expenditure profiles under which these two goals can be aligned. It provides a novel contribution to the literature through a quantitative application of the consumption corridor concept and has evaluated the resulting consumption-based energy and emission footprints.

To achieve these social goals without exacerbating energy and emission footprints, we found it necessary to reduce the expenditure of high-consuming households, affording room to those unable to achieve a minimally decent standard of living. In the next 5 years, the UK’s annual decarbonisation of territorial emissions need to accelerate from a 3% average reduction (2014–2022) to 5% between 2022 and 2030, to achieve the UK’s Nationally Determined Contribution (NDC)^52^. Increasing aggregate consumption levels to ensure minimum living standards instead of adopting explicitly redistributive policies, increases the challenge to meet the UK’s NDC, displayed by the increased footprints in the *Eliminating poverty scenario.*

By isolating the impacts of changes to the distribution of consumption across households, this study has not explored how future reductions in the GHG, or energy intensity of consumption may interact with these consumption profiles. Thus, including redistributive scenarios, minimum living standards, and their effects on production and consumption systems in energy systems modelling would develop these findings, to understand how changes in spending patterns might interact with future mitigation policies. Reducing levels of over- and under consumption are likely to yield energy and emissions benefits ceteris paribus. However, reducing levels of consumption in high energy and emissions intensity categories could accelerate this, without jeopardising the ability of households to live well. Demand side interventions that support these shifts such as increased public transport, GHG-related levies, and support for low-emission alternative provisioning are important in this regard.

Unpicking policy options to pursue this are not the focus of this study but have been explored elsewhere in the literature. Cass et al.^53^ identify four categories of policies to reduce the consumption of high energy consumers, including structural change in energy service delivery, economic disincentives, rationing approaches, and behaviour change programmes. They found that whilst some policy areas (e.g. rationing) were largely unpopular, many supported interventions to penalise their own high energy consuming activities. This was often focussed on excessive travel such as frequent flying, excess car-travel millage, or ownership of environmentally damaging vehicles. Given the particularly inequitable distribution of expenditure on transport exhibited in this study (Fig. 3), measures targeting the transport sector are likely to be distributionally just^54^, whilst also having the highest level of impact on energy and emissions footprints.

### Can consumption corridors help guide sustainable redistribution?

Reflecting on the usefulness of the consumption corridor concept considering the results of this work, the consumption corridor calculated for the UK is very narrow, given the methodological definition of the minimum and maximum consumption lines. Represented by the green area in Fig. 4, the consumption cap is only £25.99/week more than a household’s minimum consumption level, questioning its usefulness as a practical guide for a sustainable profile of consumption. This is consistent with other corridor-based redistributive studies, where near equality is also necessary to ensure minimum living standards are met alongside climate goals^15^. Whilst not a reason to avoid pursuing high levels of inequality reduction, how a consumption corridor could become more attainable is an important question.

Using a lower level of minimum consumption, for example, by adapting the decent living standards framework developed by Rao & Min^33^, and explored by others^15,31,32^ would allow the consumption corridor to be wider. However, whilst these describe the minimum necessary to meet very basic human needs, the MIS presents a fuller picture of what citizens expect to live a life with dignity in the UK.

Assuming the MIS identifies the necessary standard of living for a good life in the UK, pursuing ways through which that standard can be achieved more efficiently could widen the consumption corridor. Doing so could also have beneficial ecological and energy impacts. Increasing the access to energy efficient technologies for those beneath the MIS level of consumption, could reduce the cost, energy, and emissions implications of service provision. At present, 47.2% of ‘low income’ households in the UK were also considered to living in a low energy efficiency rated dwelling^51^. Reducing the costs required to heat homes, particularly through progressively funded public investment in home efficiency, could support the widening of the corridor whilst supporting redistribution.

Additionally, alternative provisioning systems could lower the consumption needed to reach the MIS standard of living. For example, a universal basic services (UBS) scheme could de-commodify essential goods, or resource efficiency measures like leasing schemes and the sharing economy could reduce the need for direct ownership. UBS proponents suggest that this provisioning of key services could be more economically efficient, reducing the cost of services by removing natural monopolies, transaction costs and market failures, whilst also supporting just transitions to sustainability^55^. This is supported by data on the efficiency of the housing stock, suggesting that in England, 39% of public housing fell below an EPC band C, compared with 60 and 68% of rented and privately owned housing^56^.

### Limitations and future research

Finally, it is worth considering some limitations and the avenues they present for future development of this research area. First, the study’s results are subject to the standard limitations of environmentally extended input–output (EEIO) models. Notably, a key issue for analyses of expenditure inequality is the assumption of linearity between expenditure and environmental impact. This stems from the premise that each sector in an input–output table produces a homogeneous good with a single impact vector^57^. Consequently, the model assumes, for example, that a garment costing ten times more than a comparable item has ten times the environmental impact. This simplification challenges the accurate representation of luxury or high-cost goods. Expanding and improving databases grounded in physical units would significantly advance EEIO modelling and improve assessments of consumption-based inequality and its environmental consequences. Although some models based on physical resource consumption exist^58^, studies show minimal differences between expenditure-based and physically based footprints in high-impact sectors such as fuel and aviation^11^.

Second, this study has focused on redistribution within the UK. However, whilst national redistribution is essential to a just transition to net-zero in the UK, international inequalities in consumption, as well as in contributions to climate change, are significantly wider^11,12,59^. Whilst others have investigated the climate-inequality nexus of redistributed global consumption profiles^18^, further research is essential to develop policy mechanisms for reducing international inequalities and to understand how specific countries can support redistribution between and within nations simultaneously.

A third limitation and literature gap is a lack of focus on the impacts of wealth inequality and its redistribution on transitions to net-zero. Some have explored wealth inequality and emissions^60^, possible policy options to redistribute wealth^19^, and the potential carbon impacts of a wealth tax in one European nation^61^. However, the literature has not fully explored mechanisms through which wealth redistribution could reduce ecological impacts, including those hard-to-measure, like investments, how wealth interacts with consumption habits, or how greater wealth redistribution might interact with climate policy. Understanding the ecological implications of a more equal distribution of wealth is an important avenue for future research.

A final limitation of this study, which opens opportunities for future research, is its reliance on hypothetical snapshots of final demand and the modelling of their impacts. Whilst this provides an indicative picture of the impacts relative to the three scenarios, it should not be treated as empirical data. The contribution this paper makes is a rich understanding of the energy and emissions implications of ensuring a life with dignity for all in the UK, and a description of the kind of redistributed consumption profiles that are most likely to support or hinder climate mitigation. The research would be developed further by integrating redistributive modelling into dynamic economy-environment models, which could better explain how shifts in expenditure may impact the climate transition over time, as well as how broader economic indicators would react to significant shifts in the composition of demand.

## Methods

To assess the impact of household consumption redistribution on emissions and energy, hypothetical household-type specific final demand scenarios were developed. These final demand scenarios were then modelled using a Multi-regional Input–Output model, for their energy and GHG emissions footprints, to understand how redistributing expenditure impacts the UK’s footprints. The following methodology firstly discusses the MRIO approach used to derive energy and GHG emission footprints, and then details the construction of the household consumption profiles modelled in this study.

### The UK multi-regional input–output model

An environmentally extended multi-regional input–output methodology was used to generate the energy-environment impacts of the changes made to UK final demand. Environmentally extended input–output models are a well-established in the ecological economics literature, used to assign environmental impacts to observed economic transactions, within intermediate demand (demand for products of one industry by another) and final demand (consumption of products by consumers)^62^. The Leontief equation ${\varvec{x}}={({\varvec{I}}-{\varvec{A}})}^{-1}{\varvec{y}}$ is used to determine the inter-industry purposes necessary to deliver a unit of final demand.

**Fig. 7:**
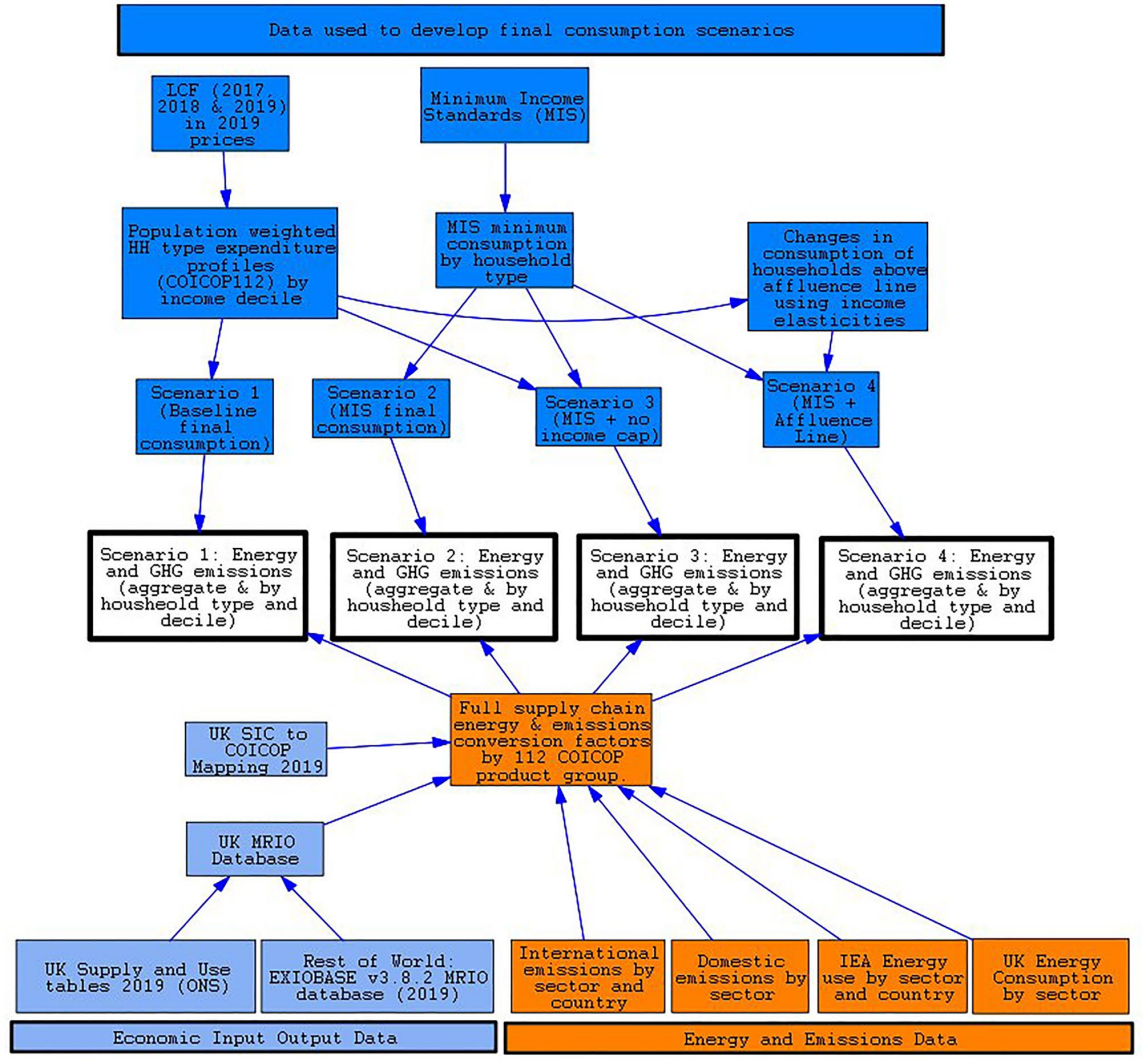
UKMRIO model framework. Source data is represented by boxes that do not have arrows going into them, whilst all boxes with arrows entering them are modelled data. Light blue represents inter-industry demand data that makes up the UK MRIO database. Orange represents the data used to environmentally extend the UKMRIO. And dark blue represents the expenditure data used to develop the final demand scenarios. UK SIC = UK Standard Industrial Classification, CIOCOP = Classification of Individual Consumption According to Purpose, IEA = International Energy Agency, LCF = Living Cost and Food Survey, UK MRIO = UK Multi-Regional Input Output model, MIS = Minimum Income Standard.

The UKMRIO model used in this study has been developed at the University of Leeds and is the source of the UK’s consumption-based emissions accounts (CBA) published annually by the UK Department for Environment, Food and Rural Affairs (DEFRA)^36^. This methodology facilitates the inclusion of emissions and energy associated with the full supply chain of goods and services consumed by households in one year^63^. Figure 7 outlines the structure of the data used in this study, with the data in dark blue, representing the construction of the final demand consumption scenarios discussed below (see "Building redistributive consumption corridor final demand scenarios."), with the light blue, and orange parts representing the data used in the construction of MRIO.

Given its use to provide the CBA to the UK Government, the UKMRIO database must be constructed using supply and use tables from the UK’s Office for National Statistics, instead of taking data on UK outputs from international MRIO databases such as EXIOBASE^64^, WIOD^65^or OECD-ICIO^66^. Inter-industry purchases from the rest of the world are taken from the EXIOBASE v3.8.2 database for the year 2019. The study uses 2019 as a base year for all data sources, as the year with the most recent final demand & impact (GHG & energy intensities) data that was not significantly affected by the COIVD 19 pandemic.

The final demand scenarios described below (see "Building redistributive consumption corridor final demand scenarios.") are sorted by COICOP categories, whereas the UK supply and use tables are in Standard Industrial Classification (SIC). Bridging tables are thus used to map expenditure by SIC to COICOP, with SIC to high-level COICOP bridging tables provided within the final demand tables of the UK’s Supply and Use Table (Table 3 HHFCe)^67^. The methodology used to disaggregate high-level COICOP to a more detailed categorisation are given in the UKMRIO model methodology^68^. This facilitates the calculation of emissions by COICOP, aligning with the final products purchased by households. To develop multipliers for the energy and emissions caused by expenditure within each COICOP category, the methodology of Owen and Buchs^69^ is used. Annual emissions and energy accounts are divided by the total weighted annual expenditure by product from the living cost and food survey. These COICOP-based emissions and energy multipliers can then be used to generate footprints based on the changes to expenditures within each COICOP category. These are described in detail in section "Building redistributive consumption corridor final demand scenarios.". For further discussion of the MRIO modelling methodology, see the supplementary note 2, as well as other publications that utilise this model^36,63,69–72^.

### Building redistributive consumption corridor final demand scenarios

To understand the energy and GHG implications of a redistributed final demand profile, several illustrative expenditure scenarios were developed that capture a snapshot of final demand over a single year. As illustrated by Fig. 2, a consumption corridor approach to sustainable redistribution was taken, ensuring everyone meets basic needs while limiting overconsumption at the top to reduce environmental impact. Figure 2 provided an overview of these scenarios, and the data processing methods and assumptions made in each scenario is discussed below.

#### Baseline scenario: current consumption inequality

The baseline scenario represents a snapshot of current consumption inequality. From this baseline, the other three final demand scenarios evaluate the extent that expenditure redistribution impacts consumption-based emissions and energy demand footprints.

The baseline case expenditure data is taken from the ONS Living Cost and Food surveys (LCF)^34^. The LCF is an annual survey of consumption practices, where each year ~ 5500 households answer questions relating to their weekly expenditure on goods and services aggregated into 112 COICOP (classification of individual consumption according to purpose) consumption categories, as well as various other descriptive statistics regarding their household. For our purposes, these include the number of adults and children living in the household, the ownership status of the dwelling, and the income of each reporting household. The individual entries to the LCF are assigned a weight, depicting the number of households each survey entry represents in wider society. As such, when all entries are multiplied by their respective weights, they come to represent an aggregate level of consumption for the UK.

To assess the distribution of consumption across different household types in this baseline scenario, the entries into the LCF are sorted into 13 categories by their household type: coupled adults with no children (C0), with one child (C1), with two children (C2) and with three children (C3), lone parents with one child (LP1), and with two children (LP2), single working age male (SWM), single working age female (SWF), male pensioners (MP), female pensioners (FP), coupled pensioners (CP), households with three adults (3A), and households with 4 adults (4A).

To avoid making inferences about household type consumption patterns from a limited dataset, three years of LCF responses were used, 2017, 2018 and 2019, ensuring that each of these household type categories contained enough entries to the survey to provide a more representative sample. These were converted into 2019 prices using the consumer price index data for each consumption category, published by the ONS^73^. These years were chosen as the latest available survey dataset that avoids the impact of the COVID-19 lockdowns on consumption levels. The selected household types represent ~ 86% of all households in the UK and were chosen due to the availability of corresponding minimum income standard budgets used to define minimum consumption levels, developed by the Joseph Rowntree Foundation^74^. As such, the presentation of results relates to this portion of UK households, rather than all households.

Households that are missing from this study are those with one parent and more than two children, omitted due to the LCF aggregating households with one parent and 3 or more children into one category. This is also the case for coupled households with 4 or more children, so they are also excluded. Other key households excluded from this analysis are those with more than 4 adults, or with more than 2 adults and the presence of children. This was due to the lack of MIS budgets for these higher occupancy household types.

Once the expenditure profiles are organised into their respective household types, the household survey entries were grouped into ten equally weighted income deciles within each household category and divided by the cumulative weight of each decile to represent the average consumption level for each household type decile.

There were some minor adjustments made to household’s expenditure on housing within the LCF, to be able to best compare inequality across levels of consumption. Given that as you go up income and consumption deciles, home ownership becomes more prevalent, housing expenditure in higher income groups is depressed, concealing the extent of inequality in consumption. This problem was corrected using an estimate of imputed rent, i.e., the market value a household would expect to pay in rent, if they did not own their house outright^75,76^. This data is not collected directly by the LCF, thus a proxy for imputed rent was calculated using the average expenditure on rent by households that are renters, for each income decile within each household type.

To arrive at footprints of the baseline scenarios, each decile’s weekly expenditures were summed to arrive at total expenditure of each of the survey entries for each household type decile. The weights assigned to each survey entry were also summed by household type decile, then these weights and the expenditure categories were divided by three, to represent just one year of data, due to the three years of LCF data included. These were then multiplied up to reach annual expenditures.

Then, each COICOP category of expenditure, was multiplied by its respective impact vector, representing the kgCO2e and kg oil equivalent per pound of expenditure, to result in total GHG emissions and total final energy use for each category. To calculate total aggregated emissions and energy use, all household type emissions or energy use was summed to create an aggregate picture.

#### Scenario 1: Minimum Income Standard scenario

The first scenario develops a hypothetical egalitarian snapshot of final expenditure where every household consumes at a level equal to the minimum level of consumption, necessary to fulfil basic needs and facilitate full participation in society. The Minimum Income Standards (MIS) produce detailed annual budgets representing a minimum level of expenditure for 11 out of 13 of the different household types explored in this study, all those listed previously, excluding households with three and four adults. For each of these 11 household types, detailed weekly baskets of goods are produced by Padley and Stone.

This expenditure was then manually sorted into the 112 COICOP groups, producing a weekly budget that could be made comparable to the LCF entries. Each household budgets is reproduce on a 4 yearly cycle to capture the dynamic nature of social norms around consumption needs, with half of the household types covered every two years on rotation. As such, five of the households’ budgets were derived in 2020 (C1, C2, C3, LP1, LP2), and six were in 2022 (SWM, SWF, C0, CP, MP, FP). These budgets were converted into 2019 prices using the consumption category specific inflation multipliers derived from the ONS consumer price index^73^. Expenditure budgets for households of three and four adults, not included in the MIS study, were found by adding 50% of the average household budget of a single working adult to the household budget of a couple, for each additional adult, as per the “OECD-modified” equivalence scale^77^.

Upon reviewing the final categorisation of the MIS budgets across the 112 consumption categories and comparing these to current compositions of demand at an economy wide scale, there were some significant differences. A decision was taken to not substantially alter the reference budgets taken from the MIS, given the value carried by the social acceptance they are prescribed due to the depth and quality of the participatory qualitative research underpinning them. However, in some situations, adjustments to better reflect current consumption preferences were made to enable these budgets to best represent consumption profiles at a nationally aggregated level. These are listed in Supplementary Information note 3. Where baseline values are taken, these are based upon the mean baseline expenditure levels within each household type. The construction of the MIS budgets for each household type are discussed in more detail in Supplementary Information note 4.

After adjustment, these household type budgets can be directly compared with baseline average consumption levels in each household type deciles, as shown in Fig. 3. To produce an aggregate consumption level where all households only consume at the socially determined minimum level necessary to meet needs and enable participation in society, the weekly expenditure of each household type MIS budget, is multiplied by the number of households in each household category, taken from the sum of the weights from each household type in the LCF dataset. These are annualised and summed together to represent an aggregate level of consumption across all household types within scope. Finally, each of the expenditure categories are multiplied by the emissions and energy use impact factors to derive footprints for energy use and emissions associated with the MIS scenario.

As discussed previously, this scenario is comparable to the ‘Reduced Consumption Scenario’ produced by Druckman and Jackson^47^, but utilises up to date budgets, reflecting current consumption preferences and social expectations for what a decent living standard looks like for UK households.

#### Scenario 2: eliminating poverty scenario

The second consumption scenario seeks to represent a case where inequality in consumption is reduced by lifting those who currently consume under their respective MIS minimum consumption level, up to that consumption level. In this scenario, no change is made to the expenditure of households consuming above this level, with the aim of evaluating the net impact of only increasing consumption of those who need to consume more, without addressing the higher levels of consumption. This is described as an *eliminating poverty scenario*, as all are now able to consume at a level that is consistent with the fulfilment of their needs and that grants them the ability to participate fully in society.

In exploring the motivations for advocating for inequality reduction within a low carbon transition, Betts-Davies et al.^21^ found that poverty reduction was often referenced across a broad spectrum of oft-opposed economic narratives of the transition, from green growth through to more radical perspectives such as post-growth and degrowth. This scenario narrative lends itself towards the former, where in green growth, poverty reduction is discussed as a key economic growth opportunity, by allowing low-income households the opportunity to participate more fully in the economy, by increasing their skills or labour productivity^21,23^. Whilst not a picture of consumption that reflects the full economic proposal of green growth, this scenario is consistent with this ideal of poverty reduction and its ability to increase final expenditure, by allowing the consumption of those below the MIS to rise, and thus growth the level of final demand in the economy.

To generate the scenario, the baseline household data from the LCF survey was used, inclusive of the adjustments described in section "Baseline scenario: current consumption inequality". Each household’s weekly consumption level was summed and compared against the respective household type’s MIS minimum consumption level. If that household fell below this level of consumption, all expenditure of that household was replaced with the expenditure profile developed by the MIS.

To arrive at a total annual level of final demand, each household’s expenditure was multiplied by its weight, and then by the number of weeks in a year (52.17857). Summing these by expenditure categories results in annual expenditures for all in scope household types. Multiplying these, by the emissions and energy impact factors described previously, results in footprints for the *Eliminating Poverty* final demand profiles.

#### Scenario 3: consumption corridor scenario

In addition to the social floor, the *consumption corridor scenario* implements a consumption cap to establish a maximum level of consumption, as per the consumption corridor framework. This cap, rather than implemented on consumption above an arbitrarily determined level, seeks to tie the level of the cap to the amount necessary to ensure a society can satisfy the needs of the population underneath the minimum consumption line. In this case, it means financial transfers from high to low consumers, equal to the amount necessary to lift those consuming below the MIS, up to that level of expenditure.

The concept is established elsewhere in the literature, first named the ‘affluence line’ by Jan Drewnowski, intended to accompany the concept of the poverty line, to address issues of social justice, resource scarcity and the ‘societal deterioration brought on by affluence’^78^. The affluence line was empirically calculated initially for the Brazilian context by Medeiros^79^, who, using a poverty line of the 33rd percentile in the Brazilian income distribution, defined the affluence line accordingly. However, the arbitrariness of this poverty line leaves it open to criticism. As a result, this scenario leans more heavily on the development made by Concialdi^75^, who, instead of using an arbitrary poverty line, utilised socially determined reference budgets (including the minimum income standard used in this paper) that define the basic needs of a society, to determine an affluence line. As a result, the definition of an affluence line borrows from Concialdi, as representing ‘the level of income above which all extra incomes would be transferred to the rest of the population in order to enable all members of the society to fully participate in it’^75^. However, given that in our case we are interested in the impacts of consumption, we use total expenditure to define this line, rather than income.

Using the social floor in consumption to define the level of maximum consumption level has some key benefits. One example is that it overcomes an ‘arbitrariness’ that could be levelled at other methods to determine a maximum consumption level, particularly given the minimum consumption level is derived through a detailed assessments of basic needs for social participation^19,75^. However, as argued by Buch-Hansen et al.^19^ the affluence line definition of a maximum income cap has its flaws, primarily in its failure to consider ecological boundaries in its evaluation of where to place the maximum income cap. However, by assessing the energy and carbon implications of this scenario, where total consumption remains stable, but where transfer payments ensure the social floor is funded, helps to offer an understanding as to the extent that redistribution can be supportive of climate mitigation, either by directly reducing emissions, or by shifting consumption into areas where decarbonisation solutions are more readily available.

##### Equivalising household consumption relative to needs

To develop a redistributed profile of final demand, that allowed all households to consume at their respective minimum consumption level, whilst facilitating the redistribution of excess consumption from one household type to another, a methodology for equivalising the consumption of different household types relative to their needs needed to be developed. Usually, economies of scale in the consumption of households are dealt with by using economic equivalence scales^75,77^, however, because reference budgets, such as those produced by the MIS, are produced directly for a specific type, these do not need to be equivalised. As such, they can act as a benchmark to compare current consumption levels with, effectively equivalising consumption levels across different household sizes.

**Fig. 8:**
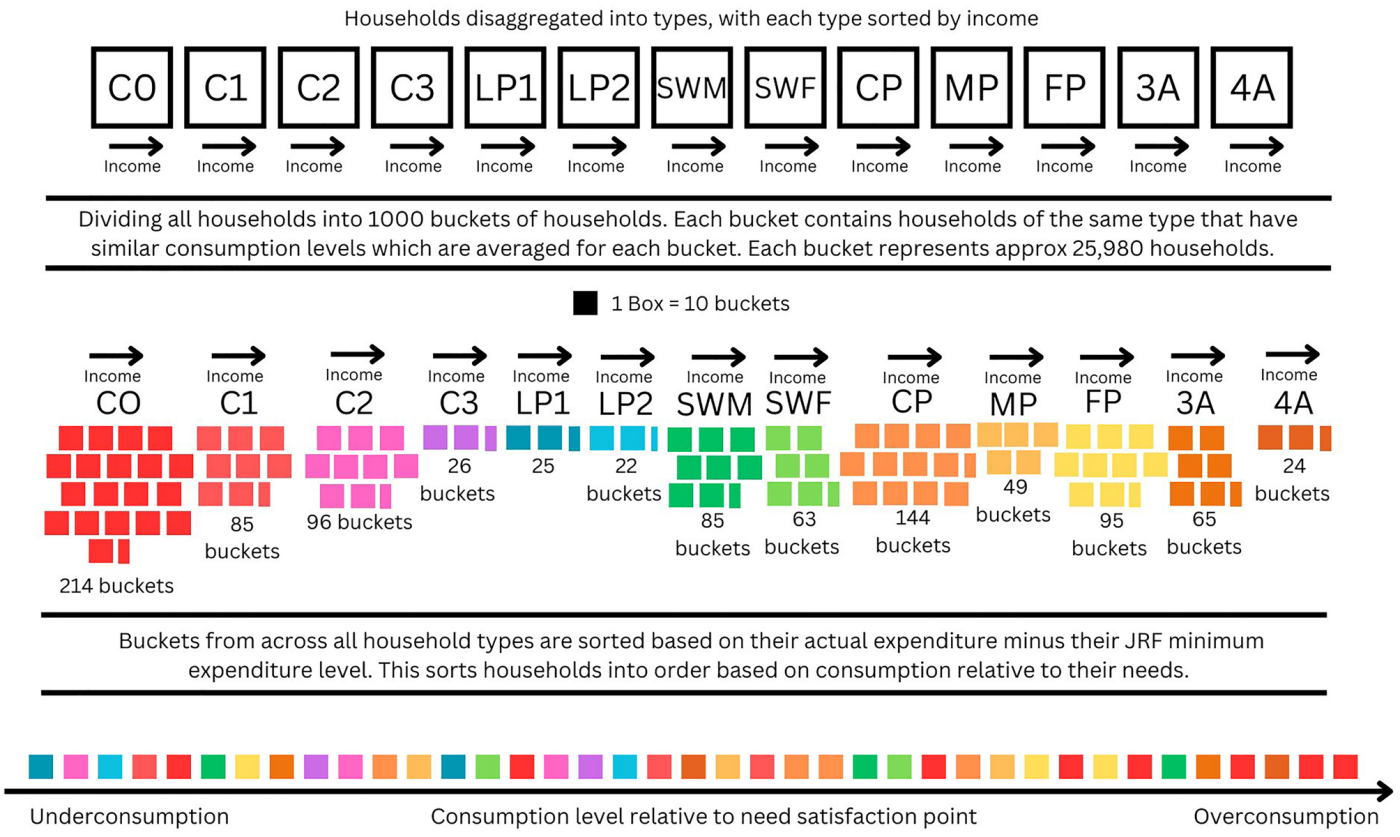
Graphical representation of methodology used to prioritise households for redistribution based on their respective needs. The ordering of buckets at the bottom of the figure is illustrative, and not indicative of the results. Household type key: coupled adults with no children (C0), with one child (C1), with two children (C2) and with three children (C3), lone parents with one child (LP1), and with two children (LP2), single working age male (SWM), single working age female (SWF), male pensioners (MP), female pensioners (FP), coupled pensioners (CP), households with three adults (3A), and households with 4 adults (4A).

This use of the household type specific minimum consumption level facilitates the prioritisation of households for redistribution based on their over consumption relative to their needs, rather than by the highest absolute consumers. Figure 8 represents this approach graphically and should be treated illustratively of the methodology, rather than precisely describing household’s ordering.

First, each of the LCF entries, adjusted as described in the baseline scenario, are sorted into their household types, and organised by order of income. Next, these entries are sorted into 1000 equally sized grouped buckets. Each bucket contains the same household type and are grouped based on the households’ similar level of consumption. In practice, this meant summing the weight of all household entries in the LCF data and dividing this by 1000 to arrive at a bucket size weight. Starting at the bottom of each household type, households were sorted into these buckets until the weights in that bucket exceeded the target bucket size weight. Within each bucket, the LCF consumption data is summed, and divided by the bucket’s respective weight, resulting in an average level of expenditure for each bucket.

After this was completed, as shown by Fig. 8, we are left with a different number of buckets for each household type, proportionate to its share of all households, with each household type’s buckets ordered by income. Within each of these buckets, we have an average level of weekly expenditure across 112 COICOP categories of consumption. Then, for each bucket, we compare the total level of expenditure to the MIS minimum level for each different household type. Subtracting total current consumption from each household’s MIS then leaves us with an indication of current consumption relative to their needs. All buckets are then ordered based on this calculation, placing the most under-consuming household at the start, and the most over-consuming household at the end. This serves to equivalise the households’ consumption relative to their respective needs.

##### Calculating the consumption cap

From this point, where all 1000 household buckets are lined up based on their current consumption level relative to their needs, the consumption cap can be calculated, following the affluence line method proposed by Medeiros^79^. The following mathematical derivation of the affluence line is thus inherited from Medeiros^79^, and adjusted for our purposes to represent consumption relative to the MIS, rather than income relative to an arbitrary poverty line.

The consumption cap **(CC)** is determined by firstly a calculation of the total need satisfaction gap necessary to ensure all are consuming at their respective MIS minimum **(NS**_***g***_**).** The ***CC*** is set at the household bucket whose consumption level is equal to the point where direct transfers in expenditure from households above which would be enough to cover ***NS***_***g***_**.** Thus, it is creating a cap where the sum of expenditure above ***CC***, the over-consumption gap **(OC**_***g***_***) is*** equal to the sum of ***NS***_***g***_ (1)*.*

1 $${OC}_{g}+{NS}_{g}=0$$

The needs satisfaction gap and overconsumption gap are found as the sum of the differences between the need satisfaction line ***(MIS)*** or consumption cap ***(CC)***, and the consumption of the lowest or highest household bucket, relative to their respective needs satisfaction line. In this distribution of consumption relative to needs, with 1000 buckets, whose unequally distributed consumption is represented by ***y***, sorted from lowest to highest, there are two groups whose consumption is adjusted relative to these two lines the ***MIS*** and **CC**. One group of high consumers, represented between ***a*** and ***b***, whose consumption is above the consumption cap, ***y***_***i***_** > CC**. And another group of low consumers between ***k*** and ***n,*** whose consumption is below the poverty line ***y***_***j***_** < JRF**. This allows Eq. (1) to be redefined as (2).

2 $$\sum_{b}^{a}\left({CC-y}_{i}\right)+ \sum_{n}^{k}(MIS- {y}_{j})=0$$

In practice, in our distribution of household buckets, ordered based on their current consumption relative to their MIS values, the ***NS***_***g***_ was found by adding all the values of those households consuming below the MIS. Given the order of households prioritises them for monetary transfers, these were assigned a value based on their order for redistribution, with the highest over consumer given the value of 1, the next 2, and so on. Then the difference between over consumer 1 and over consumer 2 is found and multiplied by the value given to it (i.e. 1), given it is only 1 bucket in the first instance that is redistributing. The next difference is multiplied by 2, given the second over consumer has joined the first in redistributing. This is then continued, adding the consumption transfer to the previous one, until the ***OC***_***g***_ is equal to the ***NS***_***g***_. This is the point at which the consumption cap is set.

##### Redistributing consumption

Once the consumption cap and need satisfaction line are calculated, the consumption profiles can be redistributed. For all buckets consuming under their MIS, this is a simple process of replacing their consumption with the consumption profile provided by the MIS, as was the case in *the eliminating poverty scenario*. However, for households consuming above the consumption cap, whilst we know how much their total consumption will reduce, we need to calculate how this will be distributed across the 112 COICOP consumption categories, to assess the impact on emissions and energy use.

As with other analyses^18,80^, elasticities were calculated to distribute the reduction of total expenditure across consumption categories. In this case, the elasticities calculated measured the percentage change of a consumption category to a 1% change in total consumption. We calculate a household type specific elasticity for each of the 13 distinct household types in this study, for 14 different product groups, mapping these onto the more disaggregated 112 COICOP groups. As done by Oswald et al^18^, our elasticities were calculated using a log–log regression of expenditure in each of the 14 aggregated categories, on total expenditure. This model was run for each household type, to produce household specific elasticities. These elasticities are reported in supplementary information table 2 below.

To test their statistical significance, the elasticities p-values were acquired, using a 95% confidence interval. Most of the elasticities calculated were deemed to be statistically significant, however there were a handful of categories for some household types where statistical significance was lacking. In these cases (as indicated by * in supplementary information table 2), the elasticities were borrowed from statistically significant results in comparable households. For example, where the lone parent with one child elasticity for clothing was an insignificant result, the elasticity was copied over from lone parent with 2 children as the closest available match.

Next, as indicated by Oswald et al.^18^, it is important to normalise the expenditure per consumption category. As these elasticities are not able to precisely allocate total consumption across our consumption categories, total expenditure when summing the adjusted expenditure categories is often higher than the reduction in expenditure dictated by the consumption cap. We thus normalise the expenditure categories so that they sum to the consumption cap. To do this, we find the share *S* of the adjusted consumption category C*i* relative to total expenditure across all consumption categories (3). Following this, adjusted category consumption (*z*) can be rescaled simply by multiplying that share by the total consumption as denoted by the consumption cap (4).

3 $${s}_{i}=\frac{{C}_{i}}{{\sum }_{i=1}^{n}{C}_{i}}$$

4 $${z}_{i}={s}_{i}*{c}_{i}$$

Applying these elasticities, and normalising consumption to equal the consumption cap determined previously leaves us with a redistributed consumption corridor, where all those consuming below the MIS are now consuming at their need’s satisfaction line, and those consuming above a level necessary to make financial transfers to afford the MIS without any increase in aggregate consumption, have been reduced to that level.

To arrive at total footprints, each of these consumption levels was multiplied by the weight of its respective bucket. Then the methodology described for producing footprints in the eliminating poverty scenario was applied to result in energy and emissions footprints for the consumption corridor scenario.

## Supplementary Information

Supplementary Information 1. Contains supplementary dataset.

Supplementary Information 2. Contains supplementary documentation as referenced in the manuscript. 

## Data Availability

The data that supports the findings of this study is available in the supplementary data section of this article, or otherwise available publicly in the locations referenced in the manuscript.
