# Supplementary material for: Quantifying the energy and emissions implications of consumption redistribution in the UK through sustainable consumption corridors
Source: Sci Rep. 2025 May 12;15:16499. doi: 10.1038/s41598-025-01495-0 (PMC12069540; doi:10.1038/s41598-025-01495-0)
Supplement: Supplementary file 2 — Supplementary Information 2. Contains supplementary documentation as referenced in the manuscript. [file 41598_2025_1495_MOESM2_ESM.docx]

# Supplementary information

Supplementary note 1: Expenditure results of redistributive scenarios

Supplementary note 2: MRIO modelling framework

Supplementary note 3: MIS scenario assumptions

Supplementary note 4: Comparing household types MIS budgets

Supplementary note 5: Consumption cap elasticities

# Supplementary note 1: Expenditure results

Figure SI1 are waterfall plots representing the changes in expenditure categories between the three scenarios relative to baseline levels of consumption. The scenarios cover a range of changes to both aggregate and the sectoral composition of expenditure. The MIS scenario represents a reduction in expenditure of 2.3%, the eliminating poverty scenario increases spending by 12.1%, and the consumption corridor remains relatively stable, with the small £0.5bn increase in expenditure only representing a 0.0008% increase in the total size of consumption.

Across all three scenarios there are some commonalities regarding sectors that have similar trends. Redistribution of expenditure, through the prioritisation of sectors as essential to ensuring a decent quality of life with dignity in the UK would see significant increases in energy costs, healthcare, and entertainment^[[1]](#footnote-1)^. Conversely, key sectors that are reduced across all three scenarios are in transport, hospitality, and household goods.

These substitution effects are largest in the MIS scenario, where the distribution of expenditure across sectors is determined explicitly by the MIS household budgets. Further these shifts represent a movement from consumption in categories with high energy and emissions intensities to those with lower ones. In the case of the MIS, whilst consumption reduces by 2.6%, the footprint savings are significantly higher at 31% for energy and 39% for emissions. This substitution is caused by shifts between categories, but also within broad categories such as transport. In contrast, the Consumption Corridor scenario, where in aggregate expenditure is only 2.4% larger than the MIS, the footprints are 50% (energy) and 60% (GHGs) larger, due to much lower levels of shifts between consumption categories.


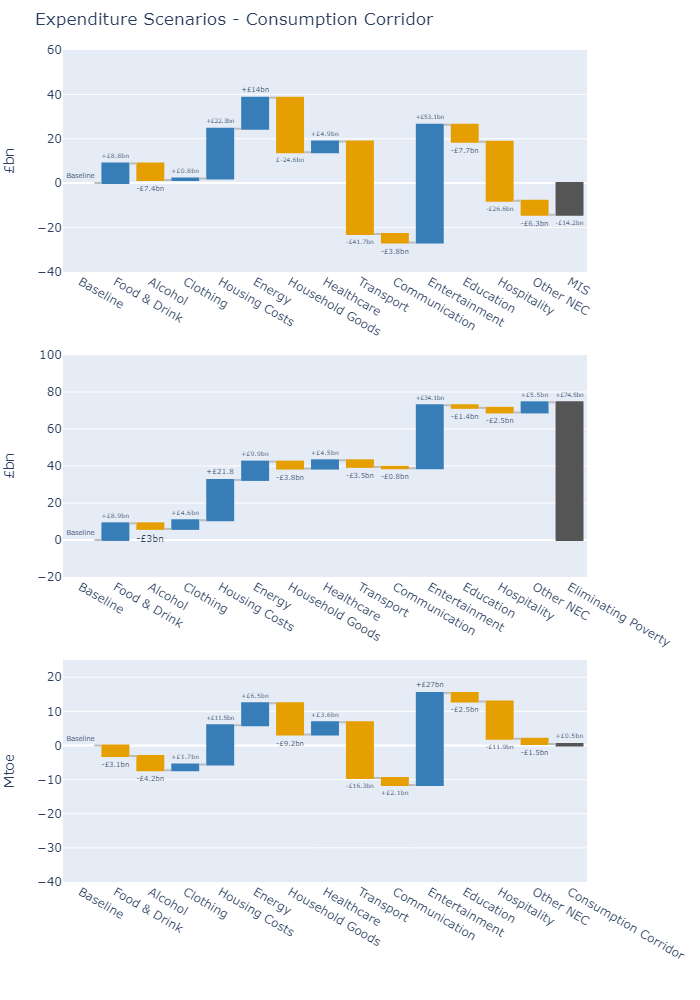


Figure SI1: Waterfall plot showing the contribution to change in the annual expenditure across high level COICOP categories based on (top) the MIS scenario, (middle) the Eliminating Poverty scenario, and (bottom) the Consumption Corridor scenario. Yellow bars represent a reduction in expenditure in a category, blue bars represent an increase, and the black bars represent total change in the expenditure within the scenario.

# Supplementary note 2: MRIO modelling framework

Figure SI 2 indicates the basic structure of the UKMRIO. Here, the transaction matrix (Z) represents intermediate demand, comprised of transactions made within and between industries. Reading across the Z matrix represents each industry’s sales, and reading down represents each industry’s purchases from other industries. Each datapoint within the Z matrix, Z_ij_, represents the contributions of a sector (i) to the industry (j). The Z matrix is comprised from adapted versions of the UK Supply and Use tables published by the ONS, for greater detail on how these are used to produce the transaction matrix, refer to Owen et al.^35^.

Y represents purchases on finished goods by final consumers and are the part of the model that is adjusted in this study, by replacing this final demand variable with hypothetical redistributive consumption scenarios as described in section 2.2. This final consumption can include spend on final products by households, as well as expenditure by government.


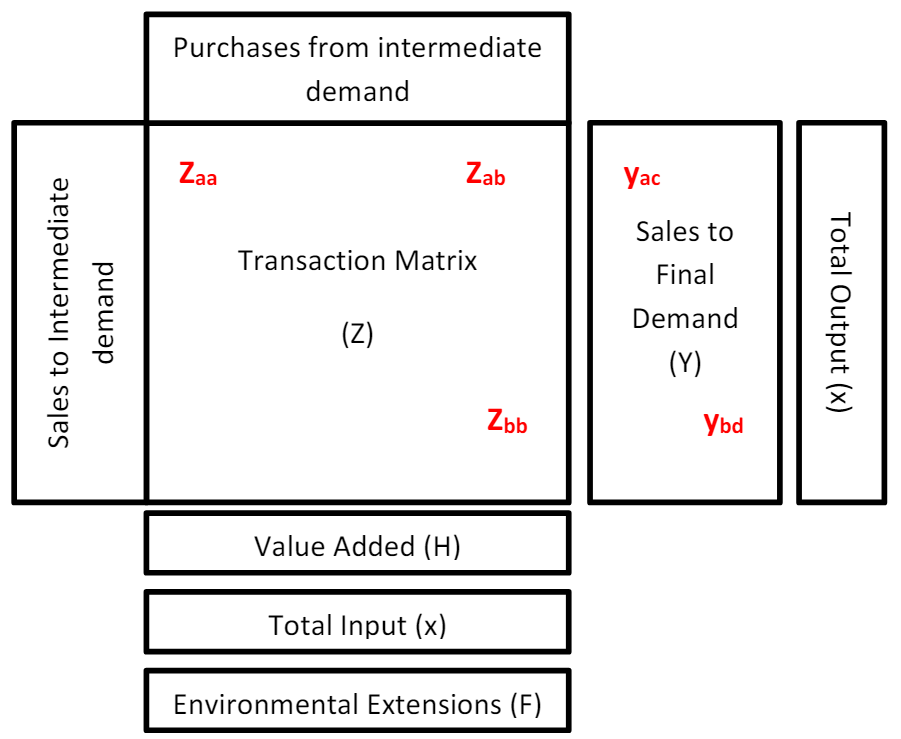


The following derivation of the UKMRIO is inherited from Owen et al. (2023).Total output for a given sector (**x_i_**) is therefore calculated as represented below, where **i** = the given sector, **j** represents intermediate sectors purchasing from sector **i**, and **Y** represents final demand for products purchased from that sector.

Figure SI 2: Basic structure of a Leontief input-output model (taken from Owen et al., 2023)

$\boldsymbol{x}_{\boldsymbol{i}}\boldsymbol{=}\boldsymbol{Z}_{\boldsymbol{i}\boldsymbol{1}}\boldsymbol{+}\boldsymbol{Z}_{\boldsymbol{i}\boldsymbol{2}}\boldsymbol{+\ldots+}\boldsymbol{Z}_{\boldsymbol{ij}}\boldsymbol{+}\boldsymbol{Y}_{\boldsymbol{i}}$ **(1)**

If we divide each element, **Z_ij_**, in row (i) by the output **x_i_** linked to the respective column (**j**), every element in **Z** can be substituted with equation 2 to form a new matrix, ***a***.

$\boldsymbol{a}_{\boldsymbol{ij}}\boldsymbol{=}\frac{\boldsymbol{Z}_{\boldsymbol{ij}}}{\boldsymbol{x}_{\boldsymbol{j}}}$ **(2)**

Inserting equation 2 into equation 1 forms:

$\boldsymbol{x}_{\boldsymbol{i}}\boldsymbol{=}\boldsymbol{a}_{\boldsymbol{i}\boldsymbol{1}}\boldsymbol{x}_{\boldsymbol{1}}\boldsymbol{+}\boldsymbol{a}_{\boldsymbol{i}\boldsymbol{2}}\boldsymbol{x}_{\boldsymbol{2}}\boldsymbol{+\ldots+}\boldsymbol{a}_{\boldsymbol{ij}}\boldsymbol{x}_{\boldsymbol{i}}\boldsymbol{+}\boldsymbol{Y}_{\boldsymbol{i}}$ **(3)**

When written in matrix notation, equation 3 is = ***Ax +Y_i_***. Solving this for ***x*** produces equation 4.

$\boldsymbol{x=}\boldsymbol{(I-A)}^{\boldsymbol{-1}}\boldsymbol{y}$ **(4)**

Here, **x** represents a vector of total output, and **Y** represents final demand. **I** is the identity matrix and ***A*** is a technical coefficient matrix which shows the inter-industry requirements. The Leontief inverse is denoted as **(I – *A*)^-1^**, hereon in identified as L in future equations. The Leontief denotes the industry-to-industry requirements of industry ***i*** to deliver a unit of output to final demand.

This economic input-output framework is then environmentally extended, to include vectors of GHG emissions generated by each sector, as well as the energy used by each sector. ***f*** represents a row vector of annual GHG emissions generated by each sector. Whilst for the purposes of this methodological description, ***f*** represents GHG emissions, it is also replaced with a vector for sectoral energy use to derive both energy and GHG footprints of the final consumption scenarios developed in this study. As such, the following formula applies to energy as well as GHG emissions when *f* is substituted for an alternative environmental extension.

$\boldsymbol{e=f}{\hat{\boldsymbol{x}}}^{\boldsymbol{-1}}$ **(5)**

***e*** is the coefficient vector that represents emissions per unit of output. Equation 6 multiplies both sides of equation 4 by ***e’*** giving:

$\boldsymbol{e}^{\boldsymbol{'}}\boldsymbol{x=e'Ly}$ **(6)**

Simplifying to:

$\boldsymbol{F=e'Ly}$ **(7)**

Here ***F*** is a matrix containing GHG emissions allowing for consumption-based emissions to be calculated. This equation shows the marginal change in GHG emissions across all sectors in response to an increase in final demand. This is calculated by pre-multiplying ***L*** by emissions per unit of output (***e’***), and post-multiplied by final demand (***Y***). Understanding this across the economy, allows the calculation of a consumption based GHG footprint.

Given that the environmentally extended IO model used in this study is one that involves various national and sub-regional regions, it is expanded to cover trade flows between international sectors. The UK MRIO covers 15 regions including: the UK, Brazil, China, India, Japan, Russia, South Africa, the USA, the EU, the rest of Europe, the rest of the OECD, the rest of Africa, the rest of the Americas, the rest of Asia and Oceania, and the Middle East, using data from EXIOBASE v3.8.2 ^61^. For full details as to how data from EXIOBASE is adapted see Owen et al. (2023).

Equation 6, expressed within the above MRIO methodology, shows the production of energy (ktoe/£) and emissions intensities (kgCO2e/£) (*e’)* per £ spent for 112 distinct consumption categories, based on tracking their expenditure from other industries in their supply chain. These can be multiplied by final expenditure within each of these categories to arrive at a consumption based GHG and energy footprint for differing footprints of final demand.

# Supplementary note 3: MIS budget adjustments

Table SI1 indicates the adjustments made to the raw MIS budgets by category to best represent consumption profiles at a nationally aggregated level.

Table SI1: Adjustments made to the raw Joseph Rowntree Foundation Minimum income standard reference budgets.

| COICOP Category | Adjustments made |
| --- | --- |
| 3.1 Clothing (3.1.1 Clothing Materials, 3.1.2 Garments, 3.1.3 Other articles of clothing and clothing accessories). | In the raw MIS budgets, all expenditure on clothing fell between COICOP category 3.1.2 and 3.1.3, excluding 3.1.1 completely. As a result, the expenditure across all three categories in the MIS was calculated and re-divided amongst these categories based on baseline proportions of consumption. The adjusted clothing budget is true to the MIS regarding the total consumption of clothing but is weighted between COICOP categories relative to shares within current consumption patterns. |
| Repair sectors (3.1.4 Cleaning repair and hire of clothing, 3.2.2 Repair and hire of footwear, 5.1.3 Repair of furniture, furnishings, and floor coverings, 5.3.3 Repair of household appliances). | Repair sectors were omitted in the MIS budgets. Therefore, we assumed that the amount of repair services demanded are proportional to the level of demand for goods that may require repair. The proportion of repair expenditure in the baseline, relative to the respective COICOP categories is taken and applied to the level of demand in the same categories in the MIS scenario. |
| Council Tax (4.4.2 Refuse collection, 4.4.3 Sewage collection, 4.4.4 Other services relating to the dwelling n.e.c.) | The MIS budgets include expenditure on council tax. The resulting expenditure on service provision isn’t modelled as part of final demand in the UKMRIO, sitting within a separate Local Authorities expenditure category. As a result, council tax expenditure is removed from the MIS budgets, and expenditure within these categories is copied over from mean household type baseline values. |
| Energy costs (4.5.1, Electricity, 4.5.2 Gas, 4.5.3 Liquid fuels, 4.5.4 Solid fuels, 4.5.5 heat energy). | The MIS budgets for household energy use are given as a single ‘fuel’ category, marking spend on all forms of household energy. This budget was initially split between heating (4.5.2 to 4.5.5) & electricity use (4.5.1) using average household bills. However, to further reflect the composition of fuel consumption in the UK at present, the heating expenditure values were split further amongst Gas (4.5.2), liquid fuels (4.5.3), solid fuels (4.5.4) and heat energy (4.5.5). This was done using the proportions of these used for household energy in 2019, as defined by the Digest of UK Energy Statistics. |
| Pharmaceutical & medical products (6.1.1 Pharmaceutical products, 6.1.2 Other medical products, 6.1.3 Therapeutic appliances and equipment). | Pharmaceutical & medical products were also recalculated to better reflect the distribution of demand amongst the COICOP categories in the baseline. The total demand for the three COICOP categories within 6.1 were redistributed amongst these categories according to the relative proportions demanded in each category in the baseline. |
| Telephone equipment and services: (8.2.1 Telephone and telefax equipment, 8.3.1 Telephone and telefax services). | In the MIS, expenditure on mobile phones was covered by a handset inclusive contract deal and was thus originally sorted into 8.3.1 (Telephone services). However, to better reflect the emissions implied by the consumption of phones in the MIS budget, an adjustment was made to split this across the two categories, by taking the retail price of the phone as a proportion of the price of the length of the contract. These factors were used to split the expenditure on mobile phones between the equipment and the service. |
| Recreational goods and services (COICOP 9.1.1 through to 9.5.4) | The MIS budgets identified expenditure on specific recreational goods, meaning many of the categories within recreational goods and services were left without any demand. As a result, unless specifically omitted in the MIS reporting (in the case of gambling (9.4.3 games of chance)), the total budget in the MIS for recreational goods and services was summed and redistributed based on the proportion of expenditure on each subcategory in the baseline. This facilitates a greater representation of current consumer choice in current consumption of recreational goods and services. |
| Restaurants and Canteens (11.1.1 Restaurants, 11.1.2 Canteens). | MIS budgets categorised all eating out expenditure within restaurants. These budgets were adjusted by taking the total spend on eating out within the MIS budget and splitting it between 11.1.1 and 11.1.2 based on the proportion of spend in the baseline. |
| Vehicle Insurance (12.5.4 Insurance connected with transport). | MIS budgets exclude the cost of vehicle insurance in this category. Therefore, the 2019 baseline data for insurance connected with transport was taken. The rate of change of demand for personal car transport between the baseline and MIS scenario was calculated and applied to the baseline figures, to reflect the reduction in car transport demanded within the expenditure on insurance. |

# Supplementary note 4: Comparing household types’ MIS budgets.

Whilst full descriptions and analyses of the MIS methodology are available elsewhere in the literature^34,43,44^, alongside full lists of the basket of goods consumed by each household type; it is still worth briefly reflecting on the MIS minimum level of consumption, and some of the key reasons why these reference budgets are useful for our purposes. Figure SI3 compares the MIS minimum consumption level across the thirteen different household types covered in this study. These household types are detailed in the legend of figure 8 and represent ~86% of UK households.

Firstly, figure SI3 indicates the breadth in needs across the different household types, both in relation to the total amount of required expenditure, as well as the distribution of consumption across categories. For example, expenditure on transport varies significantly across the different household types. In the MIS research, it was deemed necessary by UK citizens for households with children to own a car, whereas for no child households, public transport was deemed sufficient, leading to generally lower budgets for transport. One weakness of using the MIS budgets when trying to implement a minimum needs satisfaction line, is that it is unable to consider the specific needs of households beyond the household type average. As a result, there is likely to be a minority of households, of a given household type that would likely need access to a car, i.e. due to geographical or healthcare reasons, as part of their minimum needs satisfaction. This is thus a limitation inherited by this study.

Given the breadth of different household needs identified by the MIS budgets, per capita consumption minimums, such as those used in other assessments of minimum consumption levels^15,32^, are likely to obscure these key differences between household types, as well as the economies of scale in consumption gained by larger households. These are important to consider when establishing a minimum level of consumption as part of a consumption corridors approach.

Second, is an important difference in what is covered by the MIS reference budgets when compared with other much lower minimum consumption levels developed in other studies, such as the universal decent living standards framework^30–32^. The MIS budgets include relatively high expenditure in categories such as recreation, covering activities that are important for participating fully within society. Budgets for goods and services such as musical instruments for children, extra-curricular activities such as participation in sport, and attending cultural and sporting events are considered as necessary essentials for a good life within the UK context, and thus included within the MIS budgets. These are important activities that underpin quality of life, and thus should be considered when assessing the quality of life provided within sustainable futures.

Moreso, whilst the scenario in this study prescribes the MIS consumption profiles to all households, in practical application, they are used as a guide that allows for some freedom of choice over expenditure. Whilst some of the assumptions in supplementary note 3 help to represent this freedom of choice, i.e. by spready budgets on recreation across all consumption categories within that area based on current consumption trends, the MIS scenario does not allow for the individual household choice to shift expenditure from one sectoral area of expenditure to another. Thus, if implemented in practice, the footprints of an egalitarian MIS type scenario may shift relative to individual household’s consumption choices.


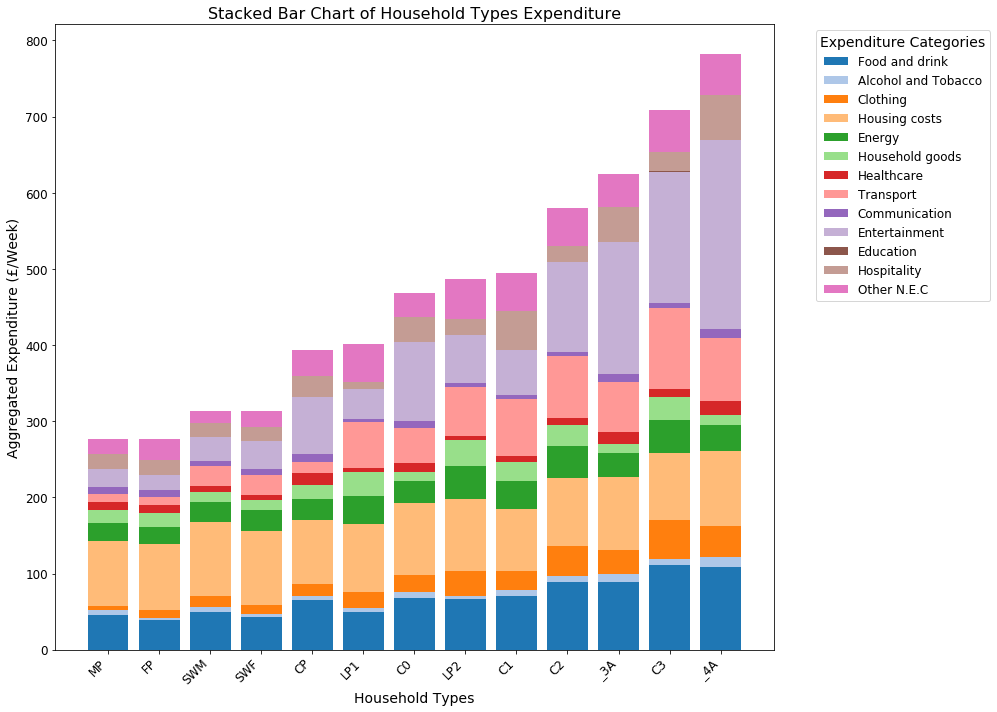


Figure SI3: Stacked bar chart displaying the Joseph Rowntree Foundation Minimum income standard household weekly expenditure profiles for different household types. Household type key: C0 = Couple, C1 = Couple + 1 child, C2 = Couple + 2, C3 = Couple + 3, LP1 = Lone parent +1 child, LP2 = Lone parent +2, MP = Male Pensioner, FP = Female Pensioner, CP = Coupled Pensioners, _3A = 3 Adults, _4A = 4 adults, SWM = Single Working Age Male, SWF = Single Working Age Female.

# Supplementary note 5: Expenditure category elasticities

Table SI2: Expenditure category elasticities relating to total expenditure. (*elasticities that have been borrowed from their closest household type due to the elasticities calculated not being statistically significant).

|  | Food | Alcohol and Tobacco | Clothing | Housing Costs | Energy | Household Goods | Healthcare | Transport | Communication | Entertainment | Education | Hospitality | NEC |
| --- | --- | --- | --- | --- | --- | --- | --- | --- | --- | --- | --- | --- | --- |
| C0 | 1.15 | 0.12 | 0.38 | 0.54 | 0.79 | 0.35 | 0.17 | 0.52 | 0.41 | 0.53 | 0.04 | 0.55 | 0.62 |
| C1 | 0.93 | 0.39 | 0.53 | 0.98 | 1.09 | 0.50 | 0.18 | 0.63 | 0.76 | 0.76 | 0.10 | 0.60 | 0.67 |
| C2 | 1.49 | 0.38 | 0.50 | 0.62 | 0.84 | 0.42 | 0.22 | 0.53 | 0.46 | 0.62 | 0.10 | 0.54 | 0.61 |
| C3 | 1.24 | 0.35 | 0.67 | 0.62* | 1.01 | 0.43 | 0.25 | 0.60 | 0.68 | 0.51 | 0.10 | 0.61 | 0.61 |
| LP1 | 1.13 | 0.35* | 0.51 | 0.76 | 0.62 | 0.33 | 0.20 | 0.55 | 0.61 | 0.71 | 0.17 | 0.66 | 0.44 |
| LP2 | 1.30 | 0.35 | 0.48 | 0.73 | 0.82 | 0.47 | 0.22 | 0.45 | 0.81 | 0.54 | 0.16 | 0.50 | 0.57 |
| MP | 1.20 | 0.39* | 0.19 | 0.74 | 0.81 | 0.33 | 0.08 | 0.41 | 0.35 | 0.52 | 0.08* | 0.53 | 0.61 |
| FP | 1.10 | 0.39* | 0.33 | 0.70 | 0.81 | 0.36 | 0.16 | 0.48 | 0.65 | 0.54 | 0.08* | 0.53 | 0.54 |
| CP | 1.61 | 0.39 | 0.41 | 0.61 | 1.08 | 0.38 | 0.21 | 0.52 | 0.40 | 0.53 | 0.08 | 0.54 | 0.63 |
| _3A | 1.08 | 0.21 | 0.41 | 0.29 | 0.58 | 0.30 | 0.10 | 0.50 | 0.48 | 0.50 | 0.08 | 0.53 | 0.64 |
| _4A | 0.77 | 0.26 | 0.42 | 0.29 | 0.63 | 0.28 | 0.20 | 0.46 | 0.54 | 0.45 | 0.08* | 0.54 | 0.70 |
| SWM | 1.20 | 0.02 | 0.19 | 0.74 | 0.81 | 0.33 | 0.08 | 0.41 | 0.35 | 0.52 | 0.02 | 0.53 | 0.61 |
| SWF | 1.10 | 0.09 | 0.33 | 0.70 | 0.81 | 0.36 | 0.16 | 0.48 | 0.65 | 0.54 | 0.08 | 0.53 | 0.54 |

1. Housing costs also display a significant increase. Some of this increase is resulting from an increased demand for suitable housing for those at the bottom end, however much of this expenditure represents the imputed rents of households who own a home. [↑](#footnote-ref-1)
